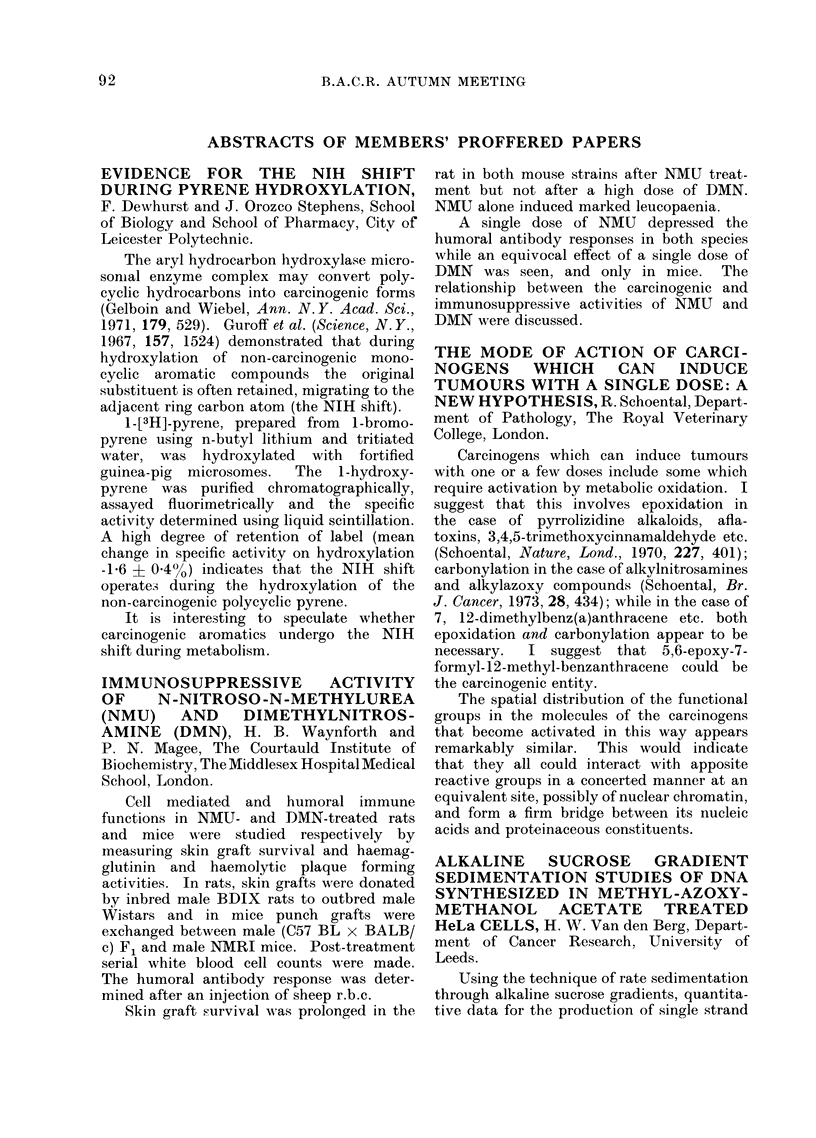# Proceedings: Immunosuppressive activity of N-nitroso-N-methylurea (NMU) and dimethylnitrosamine (DMN).

**DOI:** 10.1038/bjc.1974.14

**Published:** 1974-01

**Authors:** H. B. Waynforth, P. N. Magee


**IMMUNOSUPPRESSIVE ACTIVITY OF N-NITROSO-N-METHYLUREA (NMU) AND DIMETHYLNITROSAMINE (DMN),** H. B. Waynforth and P. N. Magee, The Courtauld Institute of Biochemistry, The Middlesex Hospital Medical School, London.

Cell mediated and humoral immune functions in NMU- and DMN-treated rats and mice were studied respectively by measuring skin graft survival and haemagglutinin and haemolytic plaque forming activities. In rats, skin grafts were donated by inbred male BDIX rats to outbred male Wistars and in mice punch grafts were exchanged between male (C57 BL $\times$ BALB/c) $F_1$ and male NMRI mice. Post-treatment serial white blood cell counts were made. The humoral antibody response was determined after an injection of sheep r.b.c.

Skin graft survival was prolonged in the rat in both mouse strains after NMU treatment but not after a high dose of DMN. NMU alone induced marked leucopaenia.

A single dose of NMU depressed the humoral antibody responses in both species while an equivocal effect of a single dose of DMN was seen, and only in mice. The relationship between the carcinogenic and immunosuppressive activities of NMU and DMN were discussed.